# Daf-2 Signaling Modifies Mutant SOD1 Toxicity in *C. elegans*

**DOI:** 10.1371/journal.pone.0033494

**Published:** 2012-03-20

**Authors:** Marco Boccitto, Todd Lamitina, Robert G. Kalb

**Affiliations:** 1 Department of Pediatrics, Division of Neurology, Children's Hospital of Philadelphia Research Institute, Philadelphia, Pennsylvania, United States of America; 2 Department of Neuroscience, University of Pennsylvania School of Medicine, Philadelphia, Pennsylvania, United States of America; 3 Department of Physiology, University of Pennsylvania, Philadelphia, Pennsylvania, United States of America; 4 Department of Neurology, University of Pennsylvania School of Medicine, Philadelphia, Pennsylvania, United States of America; Roswell Park Cancer Institute, United States of America

## Abstract

The DAF-2 Insulin/IGF-1 signaling (IIS) pathway is a strong modifier of *Caenorhabditis elegans* longevity and healthspan. As aging is the greatest risk factor for developing neurodegenerative diseases such as Amyotrophic Lateral Sclerosis (ALS), we were interested in determining if DAF-2 signaling modifies disease pathology in mutant superoxide dismutase 1 (SOD1) expressing *C. elegans*. Worms with pan-neuronal G85R SOD1 expression demonstrate significantly impaired locomotion as compared to WT SOD1 expressing controls and they develop insoluble SOD1 aggregates. Reductions in DAF-2 signaling, either through a hypomorphic allele or neuronally targeted RNAi, decreases the abundance of aggregated SOD1 and results in improved locomotion in a DAF-16 dependant manner. These results suggest that manipulation of the DAF-2 Insulin/IGF-1 signaling pathway may have therapeutic potential for the treatment of ALS.

## Introduction

ALS is an adult onset neurodegenerative disease characterized by progressive weakness, due to dysfunction and eventual death of motor neurons. The majority of cases of ALS are sporadic but single gene mutations have been described that lead to inherited versions of the disease. These genes include SOD1, TAR DNA binding protein (TDP43), fused in sarcoma, progranulin, ubiquilin 2 and a hexanucleotide repeat expansion of a noncoding region in C9ORF72 [1], [2], [3], [4], [5], [6], [7]. Expression of some of these mutant proteins in model organisms has been used to successfully model ALS pathology.

Point mutations in SOD1 (e.g., G85R) are an example of a genetic cause of familial ALS that has been successfully modeled in transgenic mice and nematodes [8]. The G85R point mutation causes a toxic gain-of-function, which in mice leads to ubiquitinated SOD1 aggregates and motor neuron death [9]. *C. elegans* expressing human G85R SOD1 in the nervous system accumulate SOD1 aggregates and demonstrate reduced mobility compared to WT SOD1 expressing worms [10]. The availability of numerous loss-of-function mutants affecting highly conserved signaling pathways make *C. elegans* an ideal system in which to explore the relationship between such pathways and SOD1 aggregation and toxicity in an *in vivo* setting.

Aging is the greatest risk factor for the development of ALS. The Insulin/IGF-1 signaling (IIS) pathway is a robust modifier of longevity and aging in *C. elegans* [11]. Loss of function of the Insulin/Insulin-like growth factor receptor, DAF-2, promotes longevity via signaling cascades mediated by inhibition of the phosphoinositide 3-kinase (*age-1*) and activation of the forkhead transcription factor DAF-16 via its nuclear localization [12]. While nuclear localization of DAF-16 is required for it to execute its transcriptional activities, it is not sufficient to enhance longevity and stress resistance [13]. Other pathways are known to interact with the IIS pathway and modulate stress resistance and/or aging by regulating transcriptional activity of DAF-16, without modifying its nuclear abundance [14]. In addition to promoting longevity, loss of function alleles of d*af-2* or *age-1* protects the worm against exogenous stressors including heat shock, oxidative stress, heavy metal stress, UV damage and infection [15], [16], [17], [18]. The beneficial effects of reduced IIS rely, in part, on the ability of decreased IIS to activate the transcription factor DAF-16, leading to increased expression of numerous stress resistance genes, such as small heat shock proteins and reactive oxygen species scavenging enzymes [18], [19]. Additionally, reduced IIS also results in changes in metabolism, mitochondrial abundance and lipid biosynthesis, all of which are thought to contribute to the stress resistant phenotype of reduced IIS [20], [21], [22]. Reduced DAF-2 signaling has been shown to have beneficial effects on other age related neurodegenerative diseases such as polyglutamine expansion proteinopathy and Alzheimer's disease [12], [23]. The ability to easily control the IIS pathway both genetically and via RNA interference (RNAi) makes the worm an excellent system for studying the interactions between aging and the toxicity of mutant SOD1. In the present study we asked if manipulation of IIS pathway can improve the reduced mobility and insoluble protein aggregation seen in G85R SOD1 expressing worms.

## Results

### Decreased crawling speed of *G85R* worms is ameliorated by decreased IIS signaling

**Figure 1:**
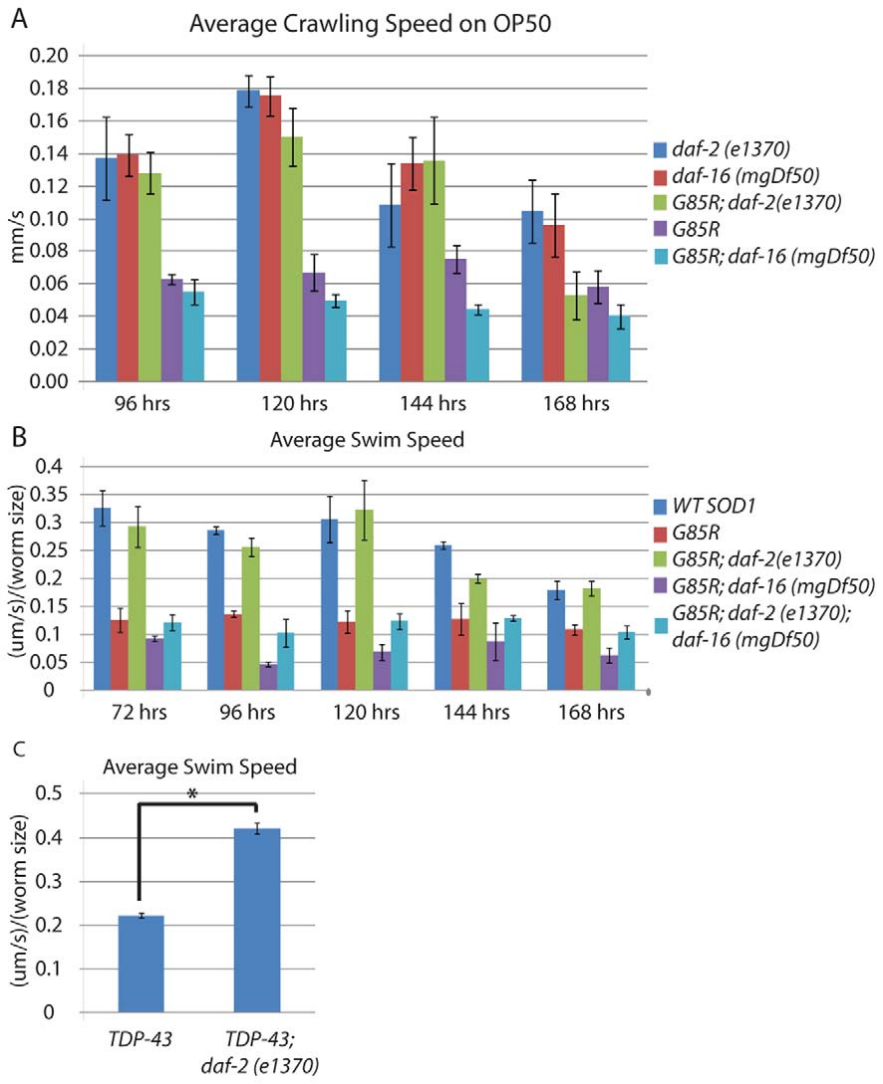
A) Videos of worms crawling on OP50 at the indicated times were taken and used to calculate worm speed using the parallel worm tracker software. B) Average swim speed normalized to size was calculated at the indicated times using the parallel worm tracker. C) Swim speed normalized to size was calculated for *TDP43* and *TDP43;daf-2(e1370)* worms 72 hrs post egg drop.

We began by monitoring the average crawling speed of *G85R, G85R;daf-2(e1370), G85R;daf-16(mgDf50), daf-2(e1370)* and *daf-16(mgDf50)* worms on a bacterial lawn (OP50) at 96, 120, 144 and 168 hours after depositing eggs onto plates with bacteria (figure 1 A). Group differences were observed at 96, 120 and 144 hrs post egg drop (96, 120 and 144 hrs: *F_(4,25)_ = 22.96, F_(4,25)_ = 11.08, F_(4,28)_ = 12.57* respectively by single factor ANOVA p<0.01 at all timepoints). At 96, 120 and 144 hrs after growth initiation, the *G85R;daf-2(e1370)* worms crawled approximately twice as fast as *G85R* worms (p<0.05 at 96, 120 and 144 hrs by Tukey's post-hoc) while at the 168 hr time point no significant difference was observed. Although not statistically significant, *G85R;daf-16(mgDf50)* worms tended to perform worse than *G85R* worms at all time points. These results suggest that reduced IIS ameliorates the toxic effects of mutant SOD1, and while this benefit is maintained for 144 hrs of life, it can not be sustained after this point. The fact that *G85R* worms that are also null for DAF-16 tend to perform worse than *G85R* worms suggests that part of the worm's endogenous response to proteotoxic insults, such as mutant SOD1, may include activation of DAF-16.

In order to control for any potential variation in locomotion and/or behavioral differences in worms with the *daf-2(e1370)* or *daf-16(mgDf50)* mutations, we examined the average crawling speed of worms carrying these mutations on a non-*SOD1* background. There were no statistically significant differences between these two groups in locomotory activity at any time point observed (by Tukey's post-hoc), suggesting that these mutations are modulating the toxicity of *G85R* as opposed to altering other aspects of behavior or locomotion in general.

### Decreased swim speed of *G85R* worms is ameliorated by decreased IIS signaling

While the crawling assay allowed us to identify an improvement in the locomotory activity of *G85R* worms when they were on the *daf-2(e1370)* background, the inability to control for behavior during a given observation window made speed measurements variable. In order to reduce variability we examined swimming worms, a context which elicits continual movement.

We compared the following worm strains in the swimming assay: *WT SOD1, G85R, G85R;daf-2(e1370), G85R;daf-16(mgDf50)*, and *G85R;daf-2(e1370);daf-16(mgDf50)* (figure 1 B). Between group differences were detected at all timepoints by single factor ANOVA (72, 96, 120, 144, and 168 hrs: *F_(4,25)_ = 15.53, F_(4,26)_ = 14.22, F_(4,25)_ = 8.67, F_(4,25)_ = 10.82, F_(4,25)_ = 9.56* p<0.01 at all timepoints). *WT SOD1* worms were significantly faster than *G85R* worms at all timepoints (p<0.01 by Tukey's post-hoc). *G85R;daf-2(e1370)* worms were also significantly faster than *G85R* worms at all timepoints (p<0.05 by Tukey's post-hoc) and had mobility equivalent to *WT SOD1* worms at all time points (no significant difference by Tukey's post-hoc). Ablation of *daf-16* in the *G85R;daf-2(e1370)* worms eliminated the observed rescue effect of *daf-2(e1370)* as no statistically significant difference was observed between *G85R* and *G85R;daf-2(e1370);daf-16(mgDf50)* worms (by Tukey's post-hoc). These observations provide further data in support of the hypothesis that the *daf-2(e1370)* background is strongly protective against the toxicity of G85R SOD1 as assessed by locomotory function and that this protection is *daf-16* dependent.

There was a trend for *G85R;daf-16(mgDf50)* worms to perform worse than either *G85R* or *(G85R;daf-2(e1370);daf-16(mgDf50)* worms although it was only significant at 96 hrs (p<0.05 by Tukey's post-hoc). The observation that *G85R;daf-16(mgDf50)* worms tended to perform worse than *G85R;daf-2(e1370);daf-16(mgDf50)* worms suggests that part of the beneficial effect of *daf-2(e1370)* may be *daf-16* independent. As in the crawling assay, the fact that *G85R;daf-16(mgDf50)* worms tended to perform worse than *G85R* worms also suggests a potential role of *daf-16* in the worm's endogenous response to proteotoxic insults. Like the crawling assay, the swimming assay further supports the hypothesis that reduced IIS activity reduces *G85R SOD* toxicity.

In order to determine the specificity of *daf-2(e1370)* on SOD1 toxicity we tested the effects of *daf-2(e1370)* on worms expressing *TDP-43* in the nervous system (Psnb-1::hTDP-43) (Figure 1 C). Like the mutant SOD1 expressing worms, transgenic expression of TDP-43 in the C. *elegans* nervous system causes locomotory defects and protein aggregation [24]. In a comparison of swim speed of *TDP-43* worms versus *TDP-43;daf-2(e1370)* worms, we found *daf-2(e1370)* improved *TDP-43* induced swimming deficit (p<0.01 by t-test).

### Improvement of the *G85R* phenotype is dependent on decreased IIS in the nervous system

**Figure 2:**
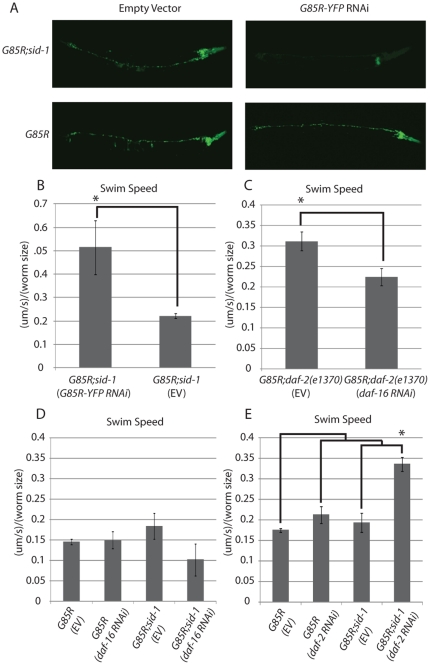
A) YFP signal was imaged in *G85R* or *G85R;unc119p::sid1;sid1(pk3321)* worms fed empty vector (EV) or G85R:YFP RNAi in order to demonstrate the efficacy of RNAi in neurons on the *unc119p::sid1;sid1(pk3321)* background B–E) Average speed normalized to size of swimming worms fed bacteria expressing the indicated RNAi.

*daf-2*(RNAi) has previously been described to mimic the longevity/healthspan promoting effects of the *daf-2(e1370)* hypomorphic allele and RNAi to *daf-16* has been shown to abrogate the benefits of loss of function of *daf-2* [25]. We next compared locomotion of *G85R* worms fed *daf-2, daf-16* or empty vector (EV) RNAi. Unexpectedly, feeding neither *daf-2* nor *daf-16* RNAi to *G85R* worms had a significant effect on locomotion (Figure 2 D,E). One possible explanation for these differences is the variable effectiveness of RNAi in the worm nervous system.

The resistance of worm neurons to RNAi can be mitigated by transgenic over expression of SID-1 in the nervous system [26]. SID-1 allows for passive cellular uptake of double stranded RNA, therefore increasing a cells response to RNAi. SID-1 is not normally expressed in neurons, therefore one way to selectively increase the nervous systems response to RNAi is through transgenic expression of SID-1 in the nervous system of worms that are null for *sid-1* in peripheral tissues. In order to maximize the efficacy of RNAi in the nervous system of *G85R* worms we generated P*snb-1::G85R::YFP; sid-1(pk3321)[Punc119::sid-1];Pmyo-6::mcherry* worms, hereafter referred to as *G85R;sid-1*. *G85R* and *G85R;sid-1* worms were fed *G85R-YFP* or empty vectro (EV) RNAi. *G85R;sid-1 w*orms fed *G85R-YFP* RNAi showed decrease YFP intensity in the nervous system and a significant (p<0.05 by t-test) increase in locomotory activity compared to *G85R;sid-1* worms fed EV RNAi (Figure 2 A–B). Feeding *G85R* worms *G85R-YFP* RNAi had no effect on fluorescence intensity or locomotory activity (Figure 2 A and data not shown). These observations lead to two important conclusions 1) they confirm the increased efficacy of neuronal RNAi in the *sid-1* nervous system expressing *sid-1(pk3321)[Punc119::sid-1];Pmyo-6::mcherry* background and 2) demonstrate that the locomotory phenotype in these worms is likely to be due to mutant G85R SOD1 expression and not integration of the transgene into a critical locus.

To better understand the role of IIS in the nervous system on G85R toxicity we compared the following groups: RNAi to *daf-2, daf-16* and EV fed to *G85R* and *G85R;sid-1* worms. While *G85R* worms fed *daf-2* RNAi showed no significant improvement in mobility compared to *G85R* worms fed EV, *G85R;sid-1* worms fed *daf-2* RNAi had significantly improved mobility compared to all other groups (ANOVA *F_(3,16)_ = 13.66* p<0.01 and p<0.01 by Tukey's post-hoc) (Figure 2 E). This disparity between the effects on *G85R* versus *G85R;sid1* worms suggests the need for decreased IIS activity in the nervous system in this model to rescue locomotory activity.

We also compared locomotion of *G85R;daf-2(e1370)* worms fed *daf-16* RNAi or EV. Feeding *G85R;daf-2(e1370)* worms *daf-16* RNAi abrogated a significant (p<.05 by t-test) amount of the *daf-2(e1370)* induced rescue of locomotory function as compared to feeding EV RNAi (figure 2 C). It is interesting that the *daf-16* (RNAi) appears to diminish *daf-2(e1370)* rescue on a non- *Punc119::sid1;sid1(pk3321)* background suggesting it is working outside of the nervous system. This raises the possibility that although reduced IIS is required in the nervous system to rescue locomotory activity in this model, there are *daf-16* dependent effects in non-nervous system tissues.

### IIS activity modulates the solubility of SOD1

**Figure 3:**
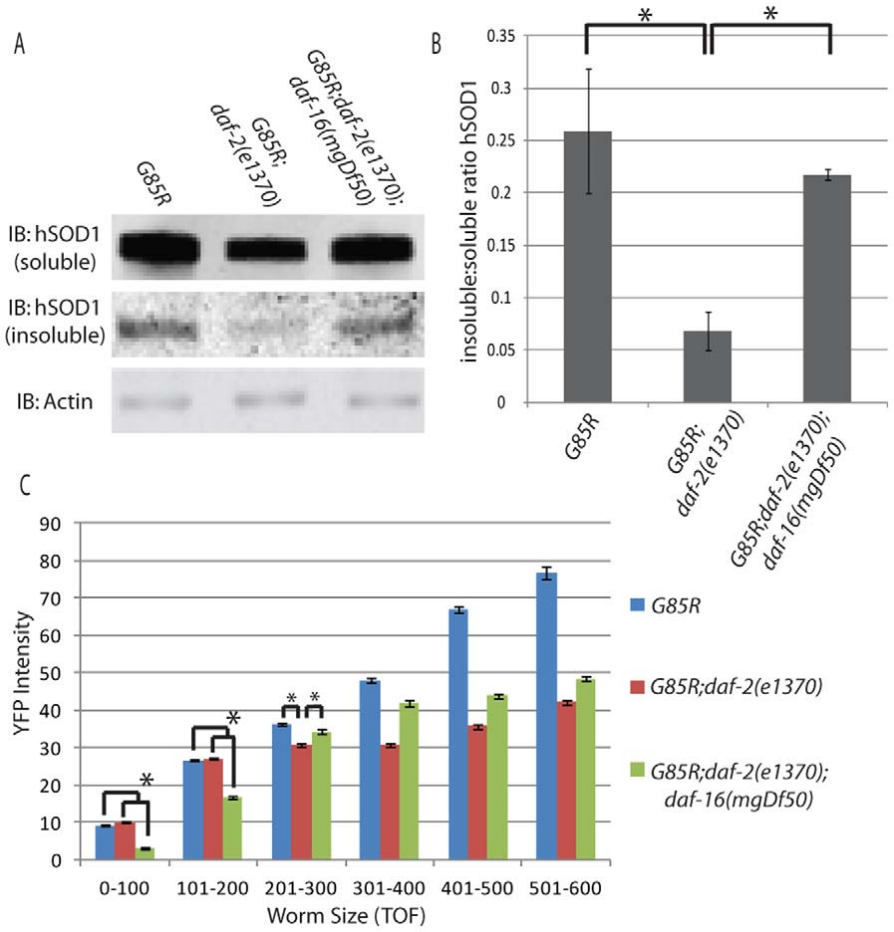
A) Representative western blot from three experiments looking at soluble vs insoluble SOD1 B) Quantification of SOD1 insoluble : soluble ratio C) COPAS data showing average YFP intensity for bins of various worm TOF.

Aging in the worm is known to be a major modifier of generalized protein solubility and aggregation, and decreased *daf-2* signaling has been found to decrease the amount of aggregation that occurs [27]. Since aggregated SOD1 generally correlates with toxicity in ALS we wanted to determine if the *daf-2(e1370)* background was decreasing aggregation in *G85R* worms. Zhang et. al. demonstrated that the *daf-2(e1370)* background significantly reduces the steady state abundance of insoluble TDP-43 in the worms we assessed in our swimming assay [24]. To determine if IIS was modifying the solubility of SOD1 in this model, soluble versus insoluble fractions were prepared from *G85R, G85R;daf-2(e1370)* and *G85R;daf-2(e1370);daf-16(mgDf50)* and immunoblotted for SOD1 and actin (figure 3A). We found a significant amount of insoluble SOD1 in the *G85R* worms which was greatly diminished in the *daf-2(e1370)* background. Deletion of *daf16* in the *G85R;daf-2(e1370);daf-16(mgDf50)* worms suppressed this effect. While the absolute values of soluble SOD1 were not constant between the three groups the ratio of insoluble to soluble SOD1 was significantly greater in the *G85R;daf-2(e1370);daf-16(mgDf50)* and *G85R* worms as compared to *G85R;daf-2(e1370)* (figure 3B) (p<.05 by single factor ANOVA, *F_(2,6)_ = 7.719* and p<0.05 for both comparisons by Tukey's post-hoc). This suggested that *daf-2(e1370)* improves the solubility of SOD1, leading to an increased steady-state abundance of the soluble species. These data were collected from a mixed population of animals so we were interested in further characterizing SOD1 abundance at various ages.

### Total SOD1 burden does not correlate with locomotor activity

While no differences in YFP intensity were evident by eye when observing the different worm strains, we wished to explore the total burden of SOD1 at various times more closely. In order to do this we used the COPAS worm sorter to measure YFP intensity and time-of-flight (TOF, i.e., length) in individual animals from a mixed population of approximately ten thousand worms from the following groups: *G85R, G85R;daf-2(e1370)* and *G85R;daf-2(e1370);daf-16(mgDf50)* (figure 3C). Using TOF as a proxy for age, we were unable to identify a direct correlation between total SOD1 burden and locomotory activity. The data were binned into TOF measurements of 100 units and significant differences between groups were observed in all bins by ANOVA (0–100 TOF *F_(2,11369)_ = 881.69*, 101–200 TOF *F_(2,7209)_ = 250.90*, 201–300 TOF *F_(2,4880)_ = 48.1*, 301–400 TOF *F_(2,3075)_ = 243.23*, 401–500 TOF *F_(2,1435)_ = 411.05*, 501–600 TOF *F_(2,708)_ = 277.65*). In young worms( 0–200 TOF) SOD1 intensity was equivalent between *G85R* and *G85R;daf-2(e1370)* worms (no significant differences between groups by Scheffe's post-hoc), yet significant locomotory differences were observed between these groups at all ages assessed using the swimming assay. Conversely, young *G85R;daf-2(e1370);daf-16(mgDf50)* worms have significantly less YFP intensity than *G85R* and *G85R;daf-2(e1370)* worms (p<0.05 by Scheffe's post-hoc), yet they perform worse than *G85R;daf-2(e1370)* worms and equivalent to *G85R* worms in our locomotory assays. Looking at older worms (300–600 TOF), *G85R* worms have a total SOD1 burden that significantly exceeds that of *G85R;daf-2(e1370);daf-16(mgDf50)* worms (p<0.05 by Scheffe's post-hoc in 301–400, 401–500 and 501–600 bins) yet they show similar locomotory activity in the swim test. This is unlikely to simply be a floor-type effect as *G85R;daf-16(mgDf50)* worms perform worse in the swimming assay than both *G85R* and *G85R;daf-2(e1370);daf-16(mgDf50)* worms, suggesting that there is room for decline in the locomotory phenotype. Taken together these results suggest that overall SOD1 burden is not responsible for the locomotory differences observed between these groups, but rather suggest that the locomotory differences result from changes in how SOD1 toxicity is handled in these various backgrounds.

### IIS effects on longevity in the SOD1 background

**Figure 4:**
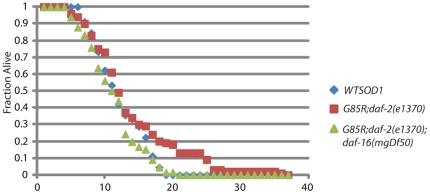
Longevity analysis, percent alive per day.

The toxicity of mutant SOD1 expression in the *C. elegans* nervous system was previously reported to have a negative effect on lifespan [10]. In order to determine whether decreased *daf-2* signaling in this background has a beneficial effect on lifespan, similar to its effect on locomotion, longevity was monitored in *WT SOD1, G85R;daf-2(e1370)* and *G85R;daf-2(e1370);daf-16(mgDf50)* worms (figure 4). *WT SOD1* and *G85R;daf-2(e1370);daf-16(mgDf50)* worms had similar lifespans, while the *G85R;daf-2(e1370)* worms had a modest but statistically significant increase in lifespan (p<0.05 by Mann-Whitney analysis). Interestingly, there is no correlation between health and lifespan as assayed by locomotory activity.

## Discussion

Aging is a common risk factor for many neurodegenerative diseases [28]. The IIS pathway is a well characterized genetic modifier of aging in *C. elegans* [11] and we demonstrate here that alterations in this pathway can robustly modify the toxicity of mutant *SOD1* as assessed by mobility, protein aggregation and longevity. We find that reduced *daf-2* activity has a beneficial effect on the toxicity of *G85R SOD1* and that this beneficial effect is *daf-16* dependent. While aging in the worm has previously been shown to be coordinated at the organismal level, we found a requirement for IIS to be reduced in the nervous system in order to modify SOD1 toxicity. Although the genetic modifiers of aging are less well characterized in humans, these data suggest that IIS and pathways which beneficially modify lifespan/healthspan in humans may be potential targets for therapeutic intervention in ALS.

Although their exact role in disease pathology is not entirely understood, aggregated proteins are associated with numerous neurodegenerative diseases, including ALS [29], [30]. As aging occurs, the total burden of aggregated proteins increases, suggesting a diminished capacity for proper folding and/or degradation of aggregation prone proteins with age [31]. In this study we demonstrate that a LOF mutation of *daf-2* diminishes the amount of insoluble SOD1; an effect that might be due to an increased capacity for clearance/folding in these worms. Similar observations have been made regarding the ability of modifications of IIS to modulate the solubility and toxicity of other disease related proteins [12], [24], . This is likely due to the ability of decreased IIS to induce expression of chaperones such as the HSP family of proteins [19]. An RNAi screen performed on these worms identified chaperones as the most highly represented functional class of proteins found to negatively modify SOD1 aggregation in this model [10]. Increased capacity for folding/clearance in worms with reduced IIS is likely to contribute to decreasing the toxcicity of mutant SOD1 in this model. We can not rule out the contribution of other changes in metabolism, lipid biogenesis, and free radical scavenger expression which have also been linked to increased lifespan and stress resistance due to decreased IIS [18], [21], [22].

Our data from the COPAS support both the concept that: 1) the *daf-2(e1370)* background can help reduce buildup of insoluble protein and 2) the ratio of soluble to insoluble G85R SOD1, rather than its total abundance, is associated with toxicity in this model. Using TOF as an approximation for age, it appears that *G85R* worms accumulate less G85R SOD1 on the *daf-2(e1370)* as opposed to wild type background over time. This suggests an increased capacity for clearance of SOD1 over time in the *daf-2(e1370)* background. The abundance of SOD1 in *G85R;daf-2(e1370);daf-16(mgDf50)* worms is less than both *G85R* and *G85R;daf-2(e1370)* in young animals yet their mobility is reduced compared to *G85R;daf-2(e1370)* and equivalent to *G85R* at all time points assessed. Although the relative abundance of SOD1 varies between these strains over time, their relative locomotory activity remains constant, suggesting that it is not total SOD1 abundance that dictates toxicity.

Previous work with a worm model of Aβ toxicity has demonstrated that the *daf-2(e1370)* allele is protective against Aβ_1–42_ aggregates in two distinct ways. First *daf-2(e1370)* led to activation of *hsf-1* which resulted in breakdown of Aβ_1–42_ fibrils. Second *daf-2(e1370)* led to activation of *daf-16* which increased the abundance of Aβ_1–42_ in high molecular weight aggregates. It is possible that both activities diminish Aβ_1–42_ toxicity by removing Aβ_1–42_from the putatively toxic fibril pool [23]. If these observations can be generalized, they could account for the lack of correlation between total SOD1 abundance and locomotory deficits in these worms. *G85R;daf-2(e1370);daf-16(mgDf50)* worms may not show the robust increase in SOD1 over time seen in the *G85R* worms because they are not accumulating the large aggregates of protein. These worms still suffer from SOD1 toxicity due to high levels of insoluble SOD1, but may never reach the same YFP intensity as *G85R* worms due to a lack of high molecular weight aggregation facilitated by *daf-16*. Activation of *hsf-1* in the *G85R;daf-2(e1370);daf-16(mgDf50)* worms may also explain their increased mobility compared to *G85R;daf-16(mgDf50)* worms in the motility assay.

Using worms with neuronal expression of SID1, we demonstrate that the beneficial effect of reduced *daf-2* is likely to be mediated by decreased IIS signaling in the nervous system. This contrasts with its effects on longevity, where *daf-2* RNAi (on a background lacking *pk3321* to enhance neuronal RNAi) mimics the lifespan extending effects of the *daf-2(e1370)* allele. In this setting, *daf-2* RNAi does not influence gene expression in the nervous system [33]. *G85R;daf-2(e1370)* worms fed *daf-16* RNAi should have normal levels of DAF-16 in their nervous system and it would be activated due to the *daf-2(e1370)* background. If the beneficial effect of decreased *daf-2* activity was completely mediated by the nervous system, then these worms should have comparable locomotory function to *G85R;daf-2(e1370)* worms fed empty vector. We find these worms to have an intermediate phenotype with a significant reduction in locomotory activity as compared to worms on empty vector RNAi plates. This suggests that part of the beneficial effect of *daf-2(e1370)* might be mediated through non-neuronal tissue(s). Alternatively, *daf-16(RNAi)* may partially, although not completely, reduce daf-16 expression in both neuronal and non-neuronal cells. Taken together, these findings suggest that although decreased IIS is required in the in the nervous system in order to have a beneficial effect on SOD1 toxicity, some of the downstream actions of reduced neuronal IIS may be functioning in the periphery.

Our results demonstrate the strong capacity of the IIS pathway to modulate G85R proteotoxicity. One possible mechanism of action for this beneficial effect is through the ability of this pathway to increase the cellular capacity to prevent toxic insoluble protein accumulation. Interestingly this beneficial activity of IIS may not be completely cell autonomous but may be in part a manifestation of alterations in cellular aging coordinated at the organismal level. This pathway may represent a possible therapeutic target for proteotoxic diseases cause by insoluble proteins.

## Materials and Methods

### Worm Strains and Handling

*C. elegans* were cultured under standard conditions at 20°C and fed the *E. coli* strain OP50 [34]. The following worm strains were used: *daf-16(mgDf50)* and *daf-2(e1370)* were obtained from the CGC. *Psnb-1::hTDP-43;Pmtl-2::GFP* and *daf-2(e1370); Psnb-1::hTDP-43;Pmtl-2::GFP* were a generous gift of Chris Link. *Psnb-1::G85R SOD1::YFP* and *Psnb-1::WT SOD1::YFP* were a generous gift from Arthur Horwich and Jiou Wang. *Punc119::sid1;sid1(pk3321)* was a generous gift from Martin Chalfie. Double and triple worms were generated by standard genetic crosses and verified by PCR or fluorescence expression.

### Locomotory Assay

Video recordings of worms were made using the image acquisition tool in Matlab 2009b. These videos were then analyzed using the parallel worm tracker software (downloaded from the Goodman lab http://wormsense.stanford.edu/tracker/). For monitoring locomotion on OP50 a 1 minute video was recorded from each plate at the center of the lawn of OP50. At 50 worms were analyzed for each genotype at each timepoint. The number of worms was greater than 30 for each genotype at each timepoint. For the swimming assay worms were suspended in a pool of M9 and their swimming was recorded for 30 seconds. At least 30 worms were analyzed per group per timepoint. Statistics were performed using the average of each video as an n of 1 for speed or size/speed.

### Feeding RNAi

HT115 bacteria containing the indicated genes in the L4440 vector were grown overnight at 37°C in 50 ug/ml ampicillin. They were then seeded on to NGM plates supplemented with 12.5 ug/ml tetracycline and 4 mM IPTG and allowed to grow overnight at room temperature. Gravid adults were allowed to drop eggs on the RNAi plates for 2 hrs and were then removed. Plates were then kept at 20°C until they were assayed.

### Imaging

Worms were immobilized in 25 mM levamisole on agar pad slides and then coverslipped. Images were acquired at a constant intensity on a confocal microscope using a 40 uM Zstack.

### Soluble vs Insoluble Protein Assay

Approximately 100 ul of packed worms were lysed via sonication in 300 ul RIPA buffer (150 mM NaCl, 50 mM Tris pH 8.0, 1 mM EGTA, 5 mM EDTA, 1% NP40, 0.5% Sodium Deoxycholate, 0.1% SDS) with complete protease inhibitor cocktail. A soft spin of 800 g for 5 min was performed to remove unlysed worms and large debris from the lysate. The supernatant was then spun at 99,000× g for 30 min @ 4°C. The supernatant was kept as the soluble fraction. The pellet was sonicated again in RIPA as a wash step to ensure removal of all soluble protein. It was centrifuged again at 99,000× g for 30 min @ 4°C. The pellet was then solublized in 50 ul urea buffer (40 mM Tris, 7 M urea, 2 M thiourea, 1% CHAPS). Equal volumes of sample were run on SDS-page gels under reducing conditions and probed with anti SOD1 antibody (Cell Signaling #2770) and anti actin (Sigma). Westerns were visualized using the Odyssey system and quantified using ImageJ.

### COPAS

COPAS was used to sort and collect fluorescence intensity from 10,000 worms from each group as described in [35].

### Longevity Assay

An egg drop was performed on NGM plates with OP50. The lifespan assay was carried out at 20°C and worms were transferred to fresh NGM OP50 plates as necessary in order to avoid starving the animals. Each day worms were counted. FUDR was not included in this assay so during the active reproductive period of the worms they were transferred to a new plate each day.
